# Synthetic surfactant with a recombinant surfactant protein C analogue improves lung function and attenuates inflammation in a model of acute respiratory distress syndrome in adult rabbits

**DOI:** 10.1186/s12931-019-1220-x

**Published:** 2019-11-06

**Authors:** J. Zebialowicz Ahlström, F. Massaro, P. Mikolka, R. Feinstein, G. Perchiazzi, O. Basabe-Burgos, T. Curstedt, A. Larsson, J. Johansson, A. Rising

**Affiliations:** 1 Division for Neurogeriatrics, Department of Neurobiology, Care Sciences and Society, Karolinska Institutet, Huddinge, Sweden; 2 Anesthesia and Intesive Care, Villa Anthea Hospital, Bari, Italy; 3 Biomedical Center Martin and Department of Physiology, Jessenius Faculty of Medicine in Martin, Comenius University in Bratislava, Martin, Slovakia; 4 Department of Pathology, The Swedish National Veterinary Institute, Uppsala, Sweden; 5 Hedenstierna Laboratory, Department of Surgical Sciences, Uppsala University, Uppsala, Sweden; 6 Department of Molecular Medicine and Surgery, Karolinska Institutet, Karolinska University Hospital, Stockholm, Sweden; 7 Department of Anatomy, Physiology and Biochemistry, Swedish University of Agricultural Sciences, Uppsala, Sweden

**Keywords:** ARDS model, Surfactant protein analogues, Synthetic pulmonary surfactant, Lung function

## Abstract

**Aim:**

In acute respiratory distress syndrome (ARDS) damaged alveolar epithelium, leakage of plasma proteins into the alveolar space and inactivation of pulmonary surfactant lead to respiratory dysfunction. Lung function could potentially be restored with exogenous surfactant therapy, but clinical trials have so far been disappointing. These negative results may be explained by inactivation and/or too low doses of the administered surfactant. Surfactant based on a recombinant surfactant protein C analogue (rSP-C33Leu) is easy to produce and in this study we compared its effects on lung function and inflammation with a commercial surfactant preparation in an adult rabbit model of ARDS.

**Methods:**

ARDS was induced in adult New Zealand rabbits by mild lung-lavages followed by injurious ventilation (V_T_ 20 m/kg body weight) until P/F ratio < 26.7 kPa. The animals were treated with two intratracheal boluses of 2.5 mL/kg of 2% rSP-C33Leu in DPPC/egg PC/POPG, 50:40:10 or poractant alfa (Curosurf®), both surfactants containing 80 mg phospholipids/mL, or air as control. The animals were subsequently ventilated (V_T_ 8–9 m/kg body weight) for an additional 3 h and lung function parameters were recorded. Histological appearance of the lungs, degree of lung oedema and levels of the cytokines TNFα IL-6 and IL-8 in lung homogenates were evaluated.

**Results:**

Both surfactant preparations improved lung function vs. the control group and also reduced inflammation scores, production of pro-inflammatory cytokines, and formation of lung oedema to similar degrees. Poractant alfa improved compliance at 1 h, P/F ratio and PaO_2_ at 1.5 h compared to rSP-C33Leu surfactant.

**Conclusion:**

This study indicates that treatment of experimental ARDS with synthetic lung surfactant based on rSP-C33Leu improves lung function and attenuates inflammation.

## Background

Acute respiratory distress syndrome (ARDS) is a serious life-threatening condition that occurs in both adults and children. Despite efforts to use different supportive and lung-protective ventilatory strategies [1, 2] the mortality in children is 20–30% [3, 4] and in adults 40% [5, 6]. ARDS may develop from direct lung injury (e.g. pneumonia, gastric contents aspiration, drowning or toxic inhalation) or secondary to extrapulmonary conditions (e.g. sepsis, hemorrhage, shock following major trauma, blood transfusion and pancreatitis) [7]. ARDS involves acute diffuse, inflammatory lung injury leading to increased pulmonary vascular permeability, and loss of aerated lung tissue. The clinical hallmarks are hypoxemia and bilateral radiographic opacities, associated with increased physiological dead space and decreased lung compliance [6]. The severity of ARDS relates to the ratio of the partial pressure of arterial oxygen (PaO_2_) to the fraction of inspired oxygen (FiO_2_) and is graded as (i) mild (P/F 200–300 mmHg, or 26.7–40 kPa), (ii) moderate (P/F 100–200 mmHg, or 13.3–26.7 kPa) and (iii) severe (P/F ≤ 100 mmHg, or ≤ 13.3 kPa) [6].

Vascular endothelium injury (caused by e.g. sepsis) and alveolar epithelium injury (e.g. following gastric content aspiration) are the most common causes of ARDS and the pathogenesis is complex. Secretory phospholipase A2 (sPLA2) activity plays an important role in the pathogenesis and can together with the oxidative environment lead to hydrolysis and oxidation of surfactant lipids [8], which results in surfactant inactivation. Also neutrophil influx and activation are of importance to the pathogenesis. The neutrophils accumulate in the lung microvasculature and become activated, leading to degranulation and the release of several toxic mediators, including proteases, reactive oxygen and nitrogen species, pro-inflammatory cytokines, and pro-coagulant molecules. This leads to loss of intercellular tight junctions as well as apoptosis and necrosis of alveolar epithelial type I and type II cells [9, 10], and subsequent increased vascular permeability and deterioration of the alveolar-capillary barrier. The protein-rich intraalveolar oedema in ARDS contains large numbers of neutrophils, monocytes, denuded epithelial cells and pro-inflammatory markers, and inactivates lung surfactant [11]. The surfactant deterioration together with oedema formation, ventilation-perfusion mismatch and inflammation lead to reduction in lung compliance and hypoxemia that further deteriorate lung function [10, 12]. Recent studies have shown that there are different phenotypes of ARDS; a more hyper-inflammatory and “un-inflamed type [13–15] and that ARDS manifests differently in different age groups. Therefore, ARDS should be divided into three different groups according to age; neonatal, pediatric, and adult ARDS [16, 17]. This increased exactness in the definition will probably both lead to improved study outcomes and, at the end, also better treatment of the patients.

ARDS patients are given supportive care, and mechanical ventilation (MV) and prone positioning are the only interventions proven to decrease mortality [1, 18]. However, the biophysical forces associated with MV might contribute to both increased inflammation and permeability, a phenomenon known as ventilator-induced lung injury (VILI) [19]. Several randomized clinical trials of exogenous surfactant therapy in adults with ARDS have been conducted [20–27], and in general show improvements in oxygenation but fail to show benefits in terms of mortality, length of intensive care unit stay or duration of mechanical ventilation [7, 28]. In addition, the results of surfactant therapy of ARDS in clinical trials have been conflicting, which may be related to variations in the surfactant composition, biophysical activity, susceptibility to inactivation, and dose [28]. Adults with ARDS probably require large amounts of exogenous surfactant, and natural surfactant preparations are prepared by laborious extraction techniques [7]. Therefore, synthetic preparations based on synthetic or recombinant proteins represent a possible alternative for generation of large quantities lung surfactant to a reasonable cost. In addition, synthetic surfactants may be more resistant to inactivation, see for example [29], but the exact reasons behind the apparent increased resistance to inactivation, for example the importance of surfactant proteins and lipids, are not established. The presence of surfactant proteins or analogues thereof is required for function of exogenous surfactant preparations in animal models of neonatal RDS [30] and are likely likewise needed in surfactant preparations intended for treatment of ARDS.

Synthetic surfactant based on recombinant surfactant protein C (SP-C) improves lung function in animal models of acute respiratory failure [31, 32]. The transmembrane poly-valyl sequence in native SP-C forms an α-helix during biosynthesis from proSP-C, but favours β-strand conformation in the absence of a functional proSP-C [33]. Therefore, SP-C analogues with a transmembrane poly-leucyl stretch have been designed to increase the α-helix propensity, avoid aggregation and facilitate production [34–36]. SP-C33Leu contains a poly-leucyl transmembrane α-helix, a positively charged residue in the N-terminal part of the helix to avoid oligomerization and a methionine residue is replaced by leucine to avoid inadvertent oxidation [37]. SP-C33Leu in phospholipid mixtures increases lung compliance similar to commercially available modified natural surfactant preparations in animal models of neonatal RDS [38]. Treatment of ARDS patients with a surfactant preparation containing recombinant human SP-C with an intact poly-valyl stretch (lusupultide) showed no mortality benefit [24, 26], but increased dose and/or changed protein and lipid composition may potentiate the clinical effect [39].

The purpose of this study was to determine the effects of treatment with recombinant SP-C33Leu (rSP-C33Leu) [40] containing surfactant compared to the animal derived surfactant poractant alfa in an experimental model of ARDS.

## Materials and methods

### Recombinant SP-C33Leu surfactant preparation

Recombinant SP-C33Leu (rSP-C33Leu) fused to the NT*-tag was expressed in a bacterial system as previously described [40]. The target peptide was released by CNBr cleavage, pelleted, resuspended in methanol:dichloroethane: H_2_O 85:10:5 (v/v/v) and purified by Lipidex chromatography (OBB, JZ, PM, JJ, TC and AR, to be published). 1,2-dipalmitoyl*-sn-*glycero-3-phosphocholine (DPPC, Sigma-Aldrich Corp., USA), L-α-phosphatidylcholine (Egg-PC, Avanti Polar Lipids, Inc., USA) and 1-palmitoyl-2-oleoyl-*sn*-glycero-3-phosphoglycerol (POPG, Sigma-Aldrich Corp., USA) were dissolved in chloroform:methanol 2:1 (v/v) and mixed in the ratio 50:40:10 (w/w/w). This phospholipid mixture togehter with SP-B and SP-C analogs gives optimal treamtent effects in a rabbit model of neonatal RDS [30]. After that 2 mg of rSP-C33Leu, dissolved in chloroform:methanol 1:1 (v/v), was added per 100 mg of the phospholipids and the mixture was dried and resuspended in physiological saline at a phospholipid concentration of 80 mg/mL to obtain a surfactant preparation containing 2% (w/w) rSP-C33Leu relative to phospholipids, herein referred to as rSP-C33Leu surfactant. The total volume of each batch was 15-45 mL depending on the number of animals that were to be treated, and it was stored at − 20 °C until use. The rSP-C33Leu surfactant was thawed in room temperature before administration.

### Animal instrumentation

The animal experiments were performed according to the ethical permit C76/16 obtained from the regional animal research committee (Uppsala Djurförsöksetiska Nämnd). Adult New Zealand white rabbits aged 15 weeks with 3.0 ± 0.3 kg body weight were used. All animals were pre-medicated with meloxicam (0.2 mg/kg; Metacam, Boehringer Ingelheim, Germany) and buprenorphine (0.07 mg/kg; Temgesic® 0.3 mg/mL, Indivior, UK) subcutaneously 30 min before anaesthesia. Subsequently, the animals were anaesthetized with ketamine (17.5 mg/kg; Ketalar® 50 mg/mL, Pfizer, Germany) and medetomidine (0.35 mg/kg; Domitor, Orion Pharma Animal Health, Sweden) intramuscularly and placed on 37 °C controlled heating surgical table in a supine position prior to the surgical procedures. The left and right marginal ear veins and left ear artery were cannulated for continuous intravenous (*i.v.*) infusion of anaesthetics (2 mL/kg/h, ketamine/xylazine in Rehydrex®), Ringer’s acetate solution (10 mL/kg/h), blood sampling and arterial pressure monitoring. Tracheotomy was performed and an endotracheal tube was inserted. After ascertained adequate anesthesia by no response to aversive stimuli of toe pinch and pinching abdominal skin with forceps, the animals were paralyzed with rocuronium bromide *i.v.* (1–2 mg/kg/h; Esmeron, Merck, USA) and the lungs were mechanically ventilated (Servoi, Maquet Critical Care, Sweden). Baseline ventilation was delivered in volume-controlled mode with a tidal volume (V_T_) of 10 mL/kg, positive end-expiratory pressure (PEEP) of 5 cm H_2_O, respiratory rate (RR) of 30 breaths per minute (bpm), inspiratory to expiratory ratio (I:E) of 1:2 and inspired oxygen fraction (FiO_2_) of 0.7 for a 30 min stabilisation period.

Monitoring included electrocardiography, invasive arterial pressure, pulse oxymetry, rectal temperature and capnography (NICO; Philips Respironics, USA). Gas exchange and parameters of acid-base balance were measured from arterial blood samples using conventional blood gas analysis (Radiometer ABL 505; Radiometer OSM3). The following parameters were calculated: P/F = calculated as the ratio between arterial oxygen partial pressure (PaO_2_) and FiO_2_; quasi-static compliance with end-inspiratory occlusion (C_stat_) = V_T_ /(P_plateau_ – PEEP); dynamic lung-thorax compliance (C_dyn_) = V_T_ /(PIP – PEEP); alveolar–arterial gradient (AaG) = [FiO_2_ (P_atm_ – PH_2_O) – PaCO_2_ /0.8] – PaO_2_, where P_atm_ is barometric pressure and PH_2_O is pressure of water vapour in the alveoli; oxygenation index (OI) = (Mean airway pressure x FiO_2_) /PaO_2_; and ventilation efficiency index (VEI) = 3800 / [(PIP – PEEP) x respiratory rate x PaCO_2_].

### Induction of experimental model of ARDS

**Fig. 1:**
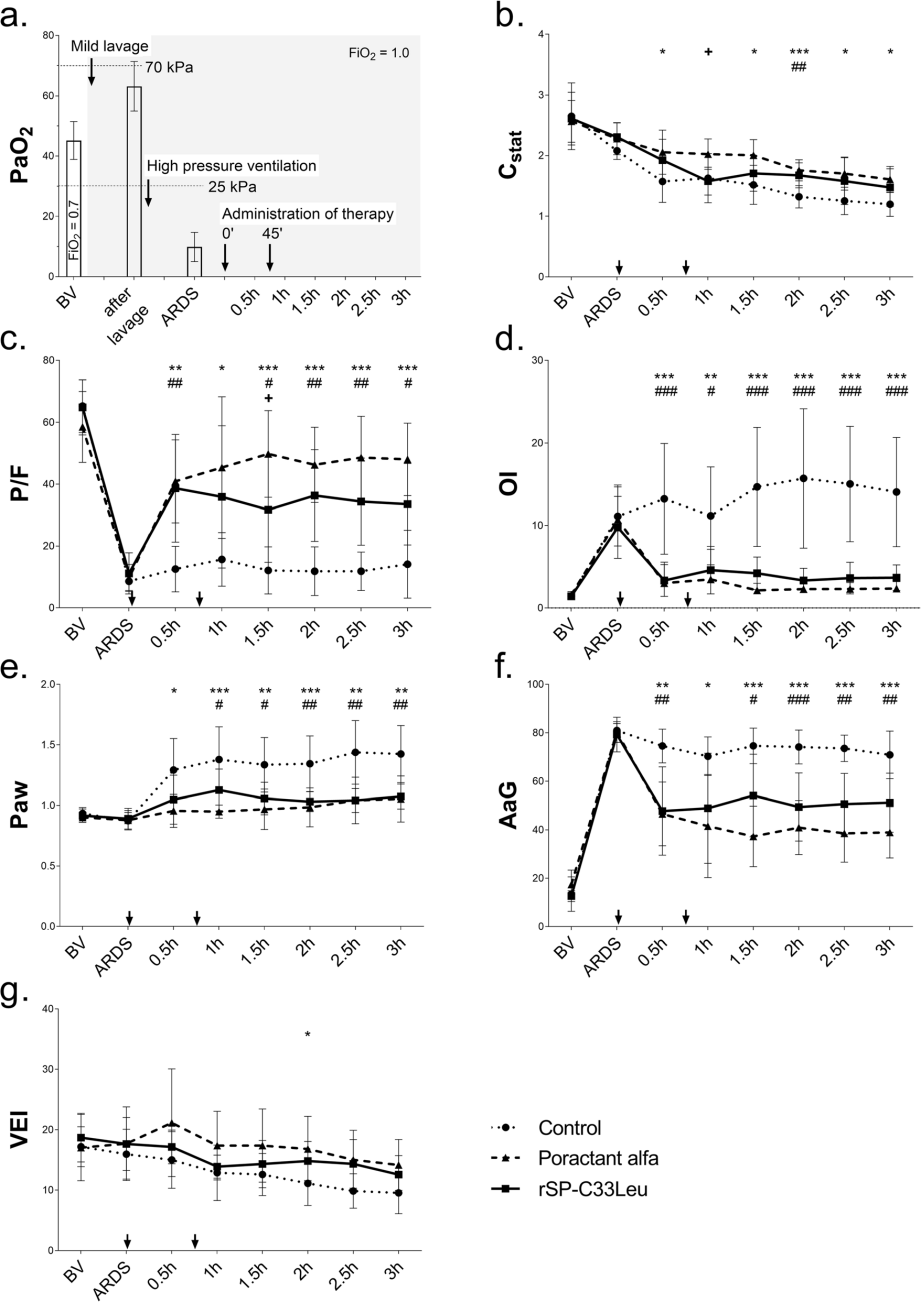
Experimental set-up and changes in respiratory parameters. (**a**) Scheme of experimental protocol and changes in PaO_2_; respiratory parameters: (**b**) staticc lung-thorax compliance (C_stat_, ml/cmH_2_O), (**c**) the ratio of arterial oxygen partial pressure to fraction of inspired oxygen (P/F, kPa), (**d**) oxygenation index (OI), (**e**) airway pressure (Paw, kPa), (**f**) alveolar-arterial gradient (AaG, kPa) and (**g**) ventilation efficiency index (VEI) before (basal value, BV) and ARDS and during 3 h after first dose of therapy. The arrows indicate administration of surfactant therapy. Data are presented as means ± SD. Statistical comparisons: for poractant alfa vs. Control ^*^p < 0.05, ^**^p < 0.01, ^***^p < 0.001; for rSP-C33Leu vs. Control ^#^*p* < 0.05, ^##^*p* < 0.01, ^###^*p* < 0.001; for poractant alfa vs. rSP-C33Leu ^+^*p* < 0.05

A two-hit model of ALI, induced by a combination of repetitive mild lung lavage and high pressure ventilation, was used in this study (Fig. 1a). After 30 min of baseline ventilation (V_T_ 10 mL/kg, PEEP 5 cm H_2_O, RR 30 bpm, I:E 1:2 and FiO_2_ 0.7), respiratory parameters and blood gases were recorded (basal values, BV). FiO_2_ was then increased to 1.0 and lung lavages were performed with warm saline (5 mL/kg, 37 °C) via the endotracheal tube. Thereafter lavage fluid was removed by lowering the tube, compression of the thorax and suction. The lung lavages were performed with the animals in supine position except the second lavage, which was performed with animals in prone position. The lavages were repeated with stabilisation periods in between (duration depending on oxygen saturation (SaO_2_)), until PaO_2_ in the arterial blood decreased to < 65 kPa in FiO_2_ 1.0. In this study, we aimed for a minimal surfactant depletion, just enough to facilitate the ARDS induction by the following injurius ventilation. Therefore the lavage volumes were very low and and the cut-off value P/F value was set very high. After that, the lungs underwent an injurious pattern of ventilation by applying a pressure-controlled mode with target V_T_:20 mL/kg, PEEP = 0 cmH_2_O, RR 20–30 bpm, I:E 1:2 and FiO_2_ 1.0. Hypocapnia was accepted without additional reduction of RR. Arterial blood gases were analysed every 0.5 h until PaO_2_ decreased to < 25 kPa, which corresponds to moderate ARDS according to the Berlin definition of ARDS.

### Treatment procedures

After the criteria of moderate ARDS were fulfilled (P/F < 25 kPa), the animals (*n* = 23) were assigned randomly to the following three groups: (i) no surfactant treatment (air bolus Control group, *n* = 7); (ii) treatment with the natural modified surfactant poractant alfa (Curosurf®, Chiesi Pharmaceutici S.p.A, Parma, Italy, *n* = 8); (iii) treatment with rSP-C33Leu surfactant (n = 8). Before treatment, a recruitment manoeuvre was performed, 6 breaths at PEEP 10 cmH_2_O and peak inspiratory pressure (PIP) 30 cmH_2_O. Surfactant treatment (2.5 mL/kg, 200 mg phospholipids/kg) was given via instillation in the trachea above carine with the animal in semi-upright right and in left lateral position (50% of the dose was given in each position). A recruitment manoeuvre with 6 additional breaths was performed after the treatment. In the control group, an air bolus (2.5 mL/kg) was given instead of surfactant. Surfactant or air delivery was repeated after 45 min using the same conditions. After treatment, the animals were placed in prone position.

After the first surfactant or air instillation, all animals were ventilated in pressure-controlled mode with a V_T_ of 8–9 mL/kg, PEEP 5 cm H_2_O, RR 25–30 bpm, I:E 1:2 and FiO_2_ 1.0 for 3 h. PEEP was increased up to 10 cm H_2_O in cases where SaO_2_ fell below 87%. PEEP was increased gradually to reach the minimum required level. Post-treatment physiological data, including blood gases and respiratory parameters were recorded every 30 min. A bolus of physiological saline (10 mL/kg) was given to animals showing hypotension. Finally, 3 h after the first treatment, the animals were euthanized under deep anesthesia by injection of potassium chloride *i.v.*. All 23 animals survived the entire protocol.

### Post-mortem tissue sampling and assays

The thorax was opened by a sternotomy with unchanged ventilation parameters. The lungs were separated from the chest and inferior vena cava and aorta were ligated and cut together with esophagus. Immediately after disconnecting the ventilator, the trachea was clamped at the carina level, and the lungs and heart were excised. Due to inherent lung heterogeneity, tissue samples from apical, medial, and caudal areas of the lungs were taken according to a pre-set scheme. Lung samples (*n* = 6 per individual) were immediately transferred into cryovials, shock frozen in liquid nitrogen and stored at − 70 °C until biochemical analyses were performed. Levels of cytokines were determined in a 10% (weight/volume) lung homogenate in 0.1 M phosphate buffer (PBS, pH 7.4). The concentrations of TNFα, IL-6, and IL-8 were quantified using rabbit-specific ELISA kits (Cloud-Clone Corp., USA) and expressed in pg/mL. The ELISA analyses were performed in duplicates, and according to the manufacturers’ instructions.

Another set of lung samples (*n* = 6 per individual) were fixed in 10% buffered formalin, embedded in paraffin, sectioned, and stained with hematoxylin-eosin. A semiquantitative analysis of interlobular and septal atelectasis/overdistension, and inflammation (dependent on oedema and leukocyte infiltration) was performed blindly by an independent pathologist (RF) and scored according to a five-graded scale: 0 = not observed, + = mild, ++ = moderate, +++ = severe, and ++++ = very severe [41]. Sum of scores were used for assessment of inflammation and atelectasis. The histological analysis of inflammatory cells was done in a semiquantitative way based on number, site, lesion distribution and extension found in two different sections. The total lung score was calculated as a sum of the scores from the apical, medial, and caudal areas of the lungs (qualitative analysis).

Extent of lung oedema was expressed as a wet-to-dry (W/D) lung weight ratio. Lung tissue samples from apical, medial, and caudal areas were weighed before and after drying in an oven at 42 °C for 2 weeks to calculate the W/D ratio.

### Statistical analysis

Statistical analysis was performed using statistical software Graph Pad Prism 6.01 (USA) and R ver. 3.5.2 with the aid of packages nlme and multcomp. All results are presented as mean ± standard deviation (SD). Data normality was tested by Shapiro-Wilk test and assessed visually by the quantile-quantile plot with the 95% bootstrap confidence band. All assessed variables except atelectasis score were distributed normally in each group – therefore we applied ANOVA for statistical analysis, one-way ANOVA with Welch’s correction in order to test the differences between the groups and one-way ANOVA with Tukey post-hoc test to test the differences between the groups in the parameters with dynamic changes for specific timepoints. Due to its non-gaussian distribution the differences in atelectasis score between treatment groups were tested by Kruskal-Wallis test. Dependence of a variable on time, within each treatment was tested within the linear mixed model, with the fixed effect of time, drug and their interaction and the random effect that allows for uncorrelated shift and slope for each animal. The same model was used to perform multiple comparisons of the means of the variable between drug treatments, using Tukey test with Benjamini Hochberg correction of the *p* values. A *p* value below 0.05 was considered to be statistically significant. The effect size corresponding to the given comparison was quantified as eta squared with values from 0 to 1.0, closer to 1.0 means larger effects. The details of statistical analysis including exact *p* values, confidence intervals (CI) and eta squared values (η^2^) are presented in Additional file 1: Table S1. The estimates of the trend (for each treatment) and *p* values using the linear mixed model with the fixed effect of time, drug and their interaction and the random effect of subjects are reported in Additional file 2: Table S2. The estimates of the difference of means and corrected *p* values using multiple comparisons of the means of a variable between drug treatments using the linear mixed model are presented in Additional file 2: Table S3.

## Results

A model of ARDS in adult rabbits that is based on mild lung lavage and subsequent high pressure ventilation was established (Fig. 1a). There were no significant differences in the baseline values (BV) of respiratory parameters between animals in the three groups (for all parameters *p* > 0.05).

### Lung function parameters

Induction of lung injury caused a severe deterioration in all measured lung function parameters; P/F ratio, oxygenation index (OI), alveolar–arterial gradient (AaG), static compliance (C_stat_), dynamic compliance (C_dyn_), and oxygen saturation (SaO_2_) were statistically significantly deteriorated at timepoint ARDS (defined as the first timepoint with P/F < 25 kPa) compared to BV, while increase in airway pressure (Paw) occurred later (Fig. 1; Table 1). When comparing the same parameter across the three experimental groups (Control vs. poractant alfa vs. rSP-C33Leu) at timepoint ARDS there were no significant differences. The deterioration of respiratory parameters persisted in the untreated control group till the end of the experiment (Fig. 1, Table 1).

**Table 1:** Respiratory parameters monitored over time. Dynamic lung-thorax compliance (C_dyn_), partial pressure of oxygen (PaO_2_), partial pressure of carbon dioxide (PaCO_2_), oxygen saturation (SaO_2_), and arterial pH before (basal value, BV) and after induced ARDS and within 3 h after administration of the therapy (Th) in two-hit ARDS untreated group (Control), and ARDS groups treated with poractant alfa or rSP-C33Leu surfactant. Data are presented as means ± SD

	BV	ARDS	0.5 h Th	1 h Th	1.5 h Th	2 h Th	2.5 h Th	3 h Th
C_dyn_ (ml/cmH_2_O)								
Control	2.2 ± 0.4	1.8 ± 0.1	1.4 ± 0.2	1.4 ± 0.3	1.3 ± 0.2	1.2 ± 0.1	1.1 ± 0.2	1.1 ± 0.2
Poractant alfa	2.3 ± 0.4	2.0 ± 0.1	1.8 ± 0.3**	1.7 ± 0.2*+	1.7 ± 0.2**	1.6 ± 0.2**	1.5 ± 0.2**	1.4 ± 0.2*
rSP-C33Leu	2.2 ± 0.5	2.0 ± 0.2	1.5 ± 0.2	1.4 ± 0.1	1.5 ± 0.2	1.5 ± 0.2*	1.3 ± 0.3	1.2 ± 0.2
PaO_2_ (kPa)								
Control	45.7 ± 6.0	8.6 ± 3.0	12.5 ± 7.4	15.7 ± 8.7	12.1 ± 5.6	11.9 ± 5.5	11.8 ± 6.2	14.1 ± 3.0
Poractant alfa	41.0 ± 8.0	9.7 ± 4.4	40.9 ± 13.4**	45.4 ± 22.9*	49.8 ± 14.0***+	46.2 ± 12.2***	48.6 ± 13.4***	48.0 ± 11.7***
rSP-C33Leu	45.4 ± 6.2	11.1 ± 6.6	38.7 ± 17.4**	35.9 ± 23.0	31.7 ± 17.0*	36.3 ± 14.8**	34.4 ± 14.1**	33.5 ± 13.2*
PaCO_2_ (kPa)								
Control	5.9 ± 1.2	4.4 ± 1.4	6.4 ± 1.1	7.3 ± 0.7	6.7 ± 0.7	7.3 ± 1.0	7.7 ± 1.9	8.1 ± 1.8
Poractant alfa	6.5 ± 2.0	4.1 ± 1.0	6.1 ± 1.2	6.5 ± 1.9	6.4 ± 1.6	6.3 ± 1.4	6.3 ± 1.4	6.5 ± 1.3
rSP-C33Leu	5.2 ± 1.4	3.8 ± 0.7	5.7 ± 0.5	5.9 ± 0.6	6.3 ± 1.3	5.9 ± 0.8	6.1 ± 1.1	6.5 ± 0.9
SaO_2_ (%)								
Control	99.0 ± 1.5	92.1 ± 7.3	94.8 ± 5.4	92.2 ± 8.5	89.4 ± 8.6	85.8 ± 11.1	87.2 ± 13.3	80.8 ± 16.8
Poractant alfa	96.9 ± 2.8	88.7 ± 6.3	96.4 ± 1.7	95.6 ± 3.6	95.6 ± 3.1	95.7 ± 2.8*	96.6 ± 1.9	96.4 ± 2.3**
rSP-C33Leu	97.3 ± 1.6	92.1 ± 5.4	96.2 ± 3.4	95.7 ± 4.3	96.5 ± 4.1	96.5 ± 4.4*	97.3 ± 3.4*	97.3 ± 3.3**
pH								
Control	7.5 ± 0.1	7.5 ± 0.1	7.4 ± 0.1	7.3 ± 0.1	7.3 ± 0.0	7.3 ± 0.1	7.3 ± 0.2	7.2 ± 0.2
Poractant alfa	7.4 ± 0.1	7.6 ± 0.1	7.4 ± 0.1	7.4 ± 0.1	7.4 ± 0.1	7.4 ± 0.1	7.4 ± 0.1	7.4 ± 0.1
rSP-C33Leu	7.5 ± 0.1	7.5 ± 0.1	7.4 ± 0.0	7.4 ± 0.0	7.3 ± 0.1	7.4 ± 0.0	7.3 ± 0.0	7.3 ± 0.0

Statistical comparisons: for Poractant alfa & rSP-C33Leu vs. Control ^*^p < 0.05, ^**^p < 0.01, ^***^p < 0.001; for Poractant alfa vs. rSP-C33Leu ^+^*p* < 0.05

Treatment with either surfactant preparation significantly improved lung function (Fig. 1, Table 1). At the first analysis after therapy (0.5 h), animals in both rSP-C33Leu and poractant alfa groups had significantly improved P/F, OI, Paw and AaG compared to the control group, and these differences persisted till the end of the experiment (Fig. 1). Poractant alfa, but not rSP-C33Leu surfactant, improved C_stat_ and C_dyn_ relative to the controls for the entire observational period and significantly improved VEI in 2 h of therapy (poractant alfa vs. Control) (Fig. 1e, f). SaO_2_ improved significantly at 2 h after the first dose in both surfactant treated groups with persisted effect till the end of experiment (Table 1). When comparing the means using the linear mixed model with the fixed effect of time, both surfactant preparation significantly improved lung function (P/F, OI, Paw, AaG, PaO_2_) compared to controls (Additional file 2: Table S3). In several key respiratory parameters (C_stat_, C_dyn_, P/F, OI, Paw, AaG, VEI), there was no statistical significant time trend present in poractant alfa and rSP-C33Leu treated groups which indicates the stability of the treatment in time (Additional file 2: Table S2).

When comparing the effects of the two surfactant therapies, no significant differences were observed for most parameters and time points. Significant differences between therapies, however, were observed in P/F ratio and PaO_2_ at 1.5 h and C_stat_ and C_dyn_ at 1 h after first dose of therapy (Fig. 1b, c, Table 1), but for some parameters poractant alfa gave more potent effect compared to rSP-C33Leu surfactant in the border of statistical significance (Additional file 1: Table S1). Significant differences of the mean between poractant alfa and rSP-C33Leu over all time points were found for C_dyn_ (*p* = 0.001) and Paw (*p* = 0.049) (Additional file 2: Table S3).

### Proinflammatory cytokines

**Fig. 2:**
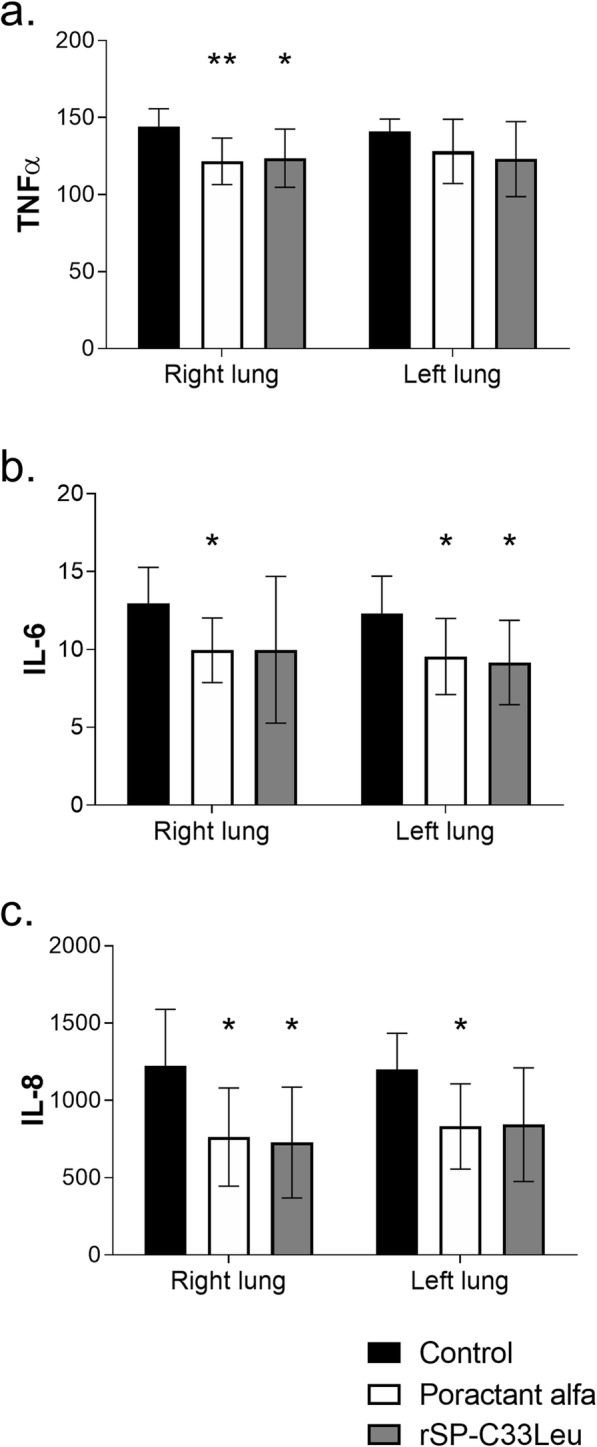
Degree of inflammation in the right and left lung. Levels of cytokines (**a**) TNFα, (**b**) IL-6 and (**c**) IL-8 (all in pg/mL) in the right and left lung tissue homogenate of untreated group (Control), and groups treated with poractant alfa or rSP-C33Leu surfactant. Data are presented as means ± SD. Statistical comparisons: for poractant alfa & rSP-C33Leu vs. Control ^*^p < 0.05

Both surfactant therapies resulted in reduced levels of IL-6 and IL-8 compared to the untreated group in both the right and left lung except rSP-C33Leu surfactant for IL-6 in right and for IL-8 in left lung (Fig. 2b and c), and reduced levels of TNFα in the right lung (Fig. 2a). When comparing the levels of cytokines in whole lungs (data now shown), poractant alfa and rSP-C33Leu surfactant treatment both resulted in decreased levels of cytokines. No statistically significant differences between the two surfactant therapies were observed, even though there was a trend that poractant alfa was more efficient (for poractant alfa: TNFα *p* = 0.002, CI: 6.91 to 28.30; IL-6 *p* = 0.002, CI: 1.13 to 4.62; IL-8 *p* = 0.004, CI: 126.6 to 579.8; and for rSP-C33Leu: TNFα *p* = 0.004, CI: 6.88 to 31.31; IL-6 *p* = 0.011, CI: 0.75 to 5.35; IL-8 *p* = 0.008, CI: 106.0 to 624.5 compared to control group).

### Histological analysis

Inflammatory cell infiltrates were found in the lung sections from animals in all three groups. In general, inflammatory cells were most prevalent in the alveoli but also were observed in blood vessels and in perivascular areas or around the airways. The inflammatory reaction was characterized by infiltrates of mixed leukocytes with a predominance of granulocytes (i.e. neutrophils and eosinophils), monocytes and macrophages, consistent with acute inflammation. The pulmonary blood vessels displayed increased number of intravascular leukocytes in a patchy distribution, which is consistent with vasculitis and ongoing migration of leukocytes into the alveolar space. Bronchioles occasionally displayed a few leukocytes in the lumen, most likely coming from adjacent inflamed alveolar ducts or alveoli.

**Fig. 3:**
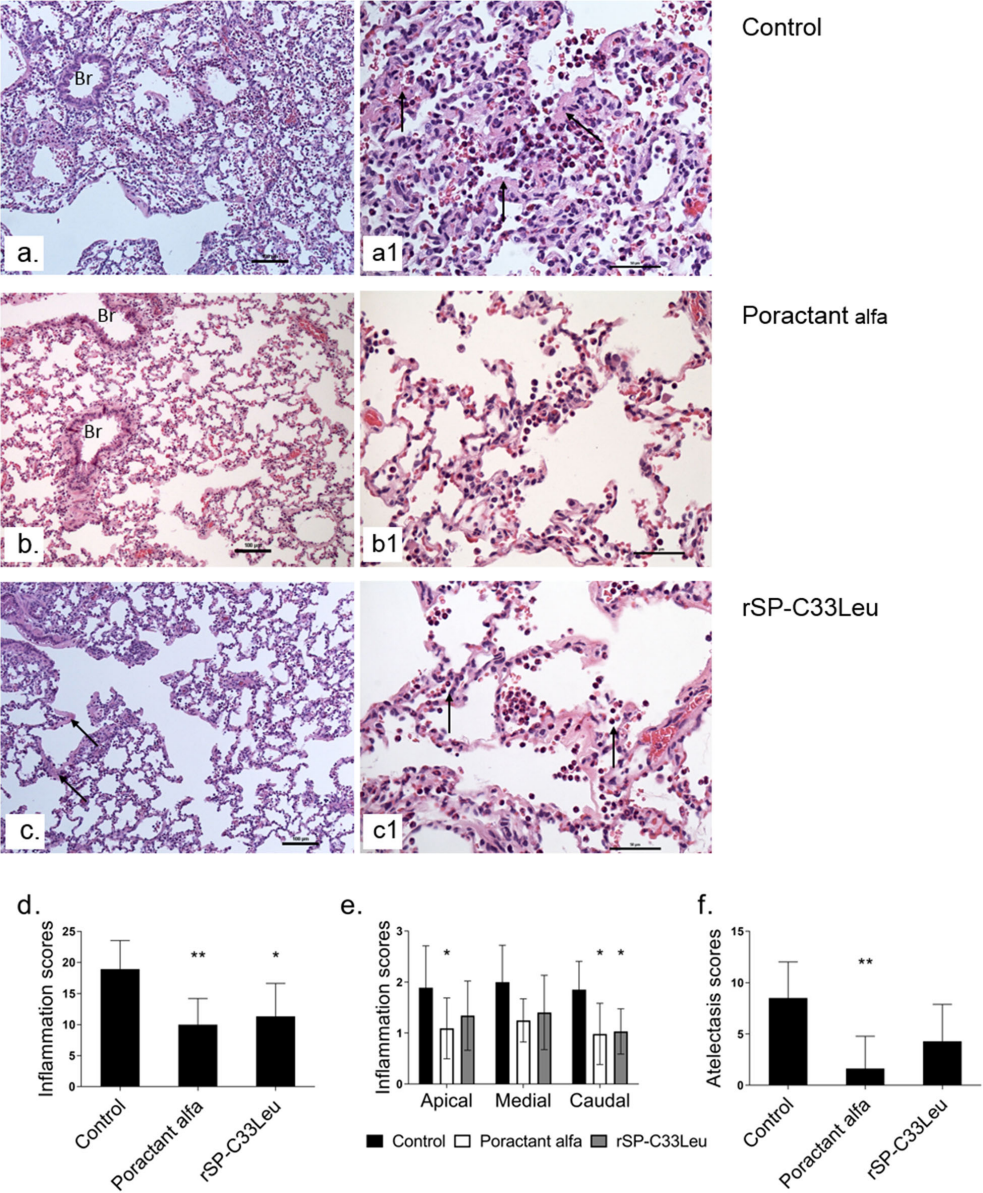
Histological analysis, inflammatory scores and atelectasis. Sections of lungs from animals in the untreated group (Control **a**, **a1**), group treated with poractant alfa (**b**, **b1**) or rSP-C33Leu surfactant (**c**, **c1**); the quantification of inflammatory response in total lungs (**d**) and apical, medial and caudal regions of lungs (**e**); and atelectasis in total lungs (**f**). In control group, pulmonary parenchyma showed a diffuse intense inflammatory cell infiltrate and collapsed alveoli (**a**). Alveolar duct and alveoli displayed an acute cell reaction of polymorphs and low numbers of macrophages. Notice the thickened alveolar septa due to deposits of a eosinophilic amorphous material (arrows) probably fibrin, a precipitate of blood proteins, or another proteinaceous material (**a1**). In the poractant alfa group, the lung seemed normal at low power, but the alveolar septa displayed slightly increased leukocytes (**b**). There were increased polymorphs and macrophages in the septal capillaries. At sites, the leukocytes have migrated into the alveolar lumens (**b1**). The lungs from the rSP-C33Leu group showed inflammatory cell reaction with leukocytes in the alveoli. Notice the thickened alveolar septa with eosinophilic material (arrows) (**c**). Alveoli showed a mild inflammatory cell reaction of granulocytes and macrophages (**c1**). Br, bronchiole. The scale bar represent 100 μm in pictures **a**, **b**, **c** and 50 μm in **a1**, **b1**, **c1**. Data are presented as means ± SD. Statistical comparisons: for poractant alfa & rSP-C33Leu vs. Control ^*^p < 0.05, ^**^p < 0.01

The inflammatory response was attenuated by both surfactant therapies compared to control animals (Fig. 3a-d) with a significant decrease in apical parts of the lungs for poractant alfa treated animals (*p* = 0.043, CI: 0.02 to 1.58) and in caudal parts for both surfactants treated groups (for poractant alfa *p* = 0.026, CI: 0.09 to 1.65; for rSP-C33Leu *p* = 0.037, CI: 0.04 to 1.60) (Fig. 3e). A significant reduction of atelectasis was observed only after the poractant alfa treatment (*p* = 0.008, CI: − 1.02 to 4.27) (Fig. 3f).

### Lung oedema formation

**Fig. 4:**
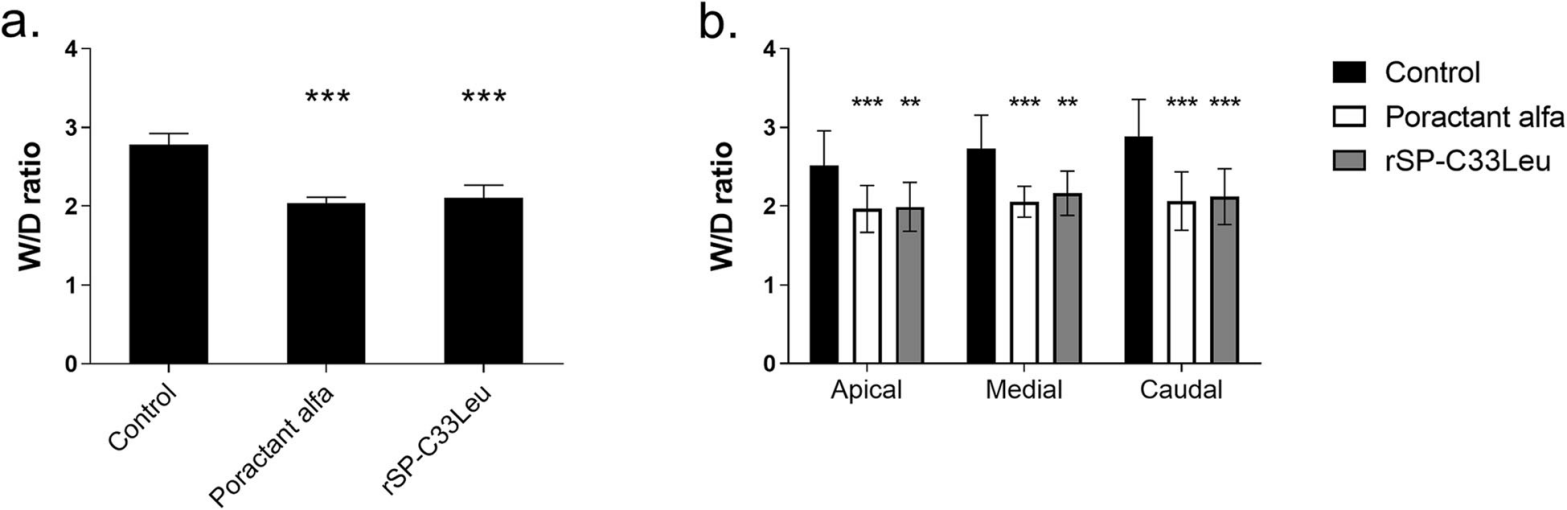
Lung oedema formation (**a**) Total lung oedema formation (**a**) expressed as wet-dry (W/D) lung weight ratio; W/D of apical, medial and caudal regions of lungs (**b**) in untreated group (Control), and groups treated with poractant alfa or rSP-C33Leu surfactant. Data are presented as means ± SD. Statistical comparisons: for poractant alfa & rSP-C33Leu vs. Control ^**^p < 0.01, ^***^p < 0.001

Degree of lung oedema was assessed by determining the wet-dry lung weight ratio (W/D ratio) of lung tissue. Several pieces of lung were collected from both lungs in each individual and used to determine the W/D ratio for the whole lung, as well as for apical medial and caudal parts. Both administration of poractant alfa (*p* = 0.0003, CI: 0.44 to 0.92) and rSP-C33Leu surfactant (*p* = 0.0008, CI: 0.53 to 0.96), respectively, significantly reduced the degree of lung oedema compared to the controls (Fig. 4a) and this was observed in all lung segments (Fig. 4b).

## Discussion

ARDS is associated with a diffuse alveolar epithelial and endothelial damage leading to an overwhelming pulmonary and systemic inflammatory response. Failure to repair the tissue damage results in a negative spiral of self-perpetuating inflammation with subsequent loss of lung function [42]. Activated sPLA2 hydrolyzes surfactant components which leads to inactivation, and plasma proteins such as albumin and fibrinogen that enter the alveolar space interfere with surfactant function and [43–45]. In addition, the inflammatory and oxidative processes could lead to type II cell apoptosis [46], and TNFα or reactive nitrogen species may directly decrease SP-A, SP-B, and SP-C synthesis in the type II cells [47, 48]. Current treatment of ARDS is mainly supportive with emphasis on management of hypoxemia. In particular, invasive mechanical ventilation with lung protective strategies is the mainstay for most ARDS patients [49]. A debated adjunctive treatment is the use of systemic glucocorticoids which may reduce inflammation and favor pulmonary repair. However, the results of randomized trials and meta-analyses are in conflict [50, 51]. In addition, several randomized clinical trials of exogenous surfactant therapy have been conducted but also these have delivered contradictory results [7, 28].

In this study we used a combination of repeated small volume lung lavage with saline and high-pressure ventilation to induce ARDS in adult rabbits. Since the therapeutic intervention was tracheal surfactant instillation, this particular model was carefully designed to avoid any significant surfactant depletion by the lavage. The purpose of this minimal lavage was to prime for the inflammatory lung injury (causing chemical surfactant inactivation) created by the following high-pressure lung ventilation. The high lung volumes lead to alveolar rupture, air leakage and regional lung overdistension [19]. This model should be particularly relevant for VILI or ARDS that develops in patients that are ventilated for other lung injuries. On the other hand, it may not be as good for studying indirect ARDS or ARDS that develops secondary to aspiration of e.g. meconium, milk or gastric contents. Future studies of artificial surfactant preparations should therefor include complimentary models of ARDS where other aspects of the condition are better recapitulated.

After induction of ARDS and administration of surfactant, the animals were placed in prone position to increase homogeneity of ventilation [19, 52]. About 70% of patients with ARDS and hypoxemia have improved oxygenation in a prone position, from increased end-expiratory lung volume, better ventilation–perfusion matching, less effect of the mass of the heart on the lower lobes, and improved regional ventilation [53]. Despite these measures the respiratory parameters of the control animals remained deteriorated until the end of experiment, which indicates that we have established a stable model of ARDS. The two-hit intervention caused the parameters P/F, OI and AaG to deteriorate within minutes, in line with what has previously been shown [54–56]. Subsequently, surfactant therapy was administered as a bolus two times, the second bolus 45 min after first dose, in an effort to overcome potential surfactant inactivation. Treatment with poractant alfa or rSP-C33Leu surfactant improved lung function parameters to a similar extent. Rapid improvement in P/F, OI and AaG compared to the control animals was observed within the first 30 min after administration and persisted till the end of experiment, which indicate that both surfactant preparations were active and improved the gas exchange. These results are similar to previous studies of surfactant therapy in ARDS models, where rapid improvements in arterial oxygenation and/or lung mechanics have been seen [57–60]. Poractant alfa improved respiratory parameters to a larger extent compared to rSP-C33Leu surfactant and significant differences between the surfactant therapies were observed at some timepoints for P/F, PaO_2_ and C_dyn_ (Fig. 1, Table 1, Additional file 1: Table S1). This may be due to the lack, in the rSP-C33Leu surfactant, of an SP-B analogue and/or to the different phospholipid composition in comparison to poractant alfa. In some respiratory parameters, a slight deterioration was observed after the second surfactant bolus and this was more obvious after administration of rSP-C33Leu surfactant (Fig. 1). This may be an effect of increased fluid volume in the lungs and/or be a consequence of that rSP-C33Leu surfactant was somewhat more viscous than poractant alfa.

In response to lung injury, there is a massive influx of leukocytes especially neutrophils from the circulation into the interstitium and alveolar spaces [61]. In accordance, infiltrates of granulocytes (i.e. neutrophils and eosinophils), monocytes and macrophages were observed in histological lung sections at 3 h after ARDS induction. Activation of these cells is associated with production of pro-inflammatory cytokines such as TNFα, IL-6 and IL-8 [54, 55, 62]. Inflammatory mediators and bioactive substances including reactive oxygen and nitrogen species damage the endothelial and epithelial cells and thereby increase the permeability across the alveolar-capillary membrane, resulting in pulmonary oedema formation [9, 63]. It is therefor important to mitigate the inflammatory response early in the treatment of ARDS. In our experiments, both poractant alfa and rSP-C33Leu surfactant treatments reduced the level of pulmonary inflammation and resulted in decreased lung oedema compared to the control group (Figs. 2, 3 and 4). In this study we focused on inflammatory markers and did not analyze specific markers for surfactant dysfunction, endothelial damage or oxidative stress. These limitations should be addressed in future studies. In particular, analysis of sPLA2 could be informative since it links inflammation and surfactant dysfunction, and correlates with clinical outcomes in ARDS patients [64–66].

Clinical trials of surfactant therapy in adults with ARDS generally have shown improvements in oxygenation indices but the results have to some degree been contradictory and failed to produce any demonstrable survival benefits [22, 24, 26, 67]. This could be due to heterogeneity of the patient populations, dose of surfactant given, and surfactant composition [7, 28]. The resistance to inhibition of the exogenous surfactants could depend on the concentrations of SP-B and SP-C (or their analogues) as well as the phospholipid composition. In a premature rabbit foetus model of neonatal RDS phospholipid composition is important for tidal volume while the SP-B and SP-C analogues increase alveolar stability at end-expiration [30]. Compared with endogenous surfactant the animal derived surfactant preparations contain lower and different amounts of SP-B and SP-C [68]. Furthermore, synthetic surfactants containing recombinant SP-C or a leucine/lysine polypeptide are in vitro more resistant to inhibition by meconium components than the modified natural surfactants [69]. This suggest that recombinant SP-C, or analogues thereof, can be used as a starting point for the design of new surfactant formulations that are more resistant to inactivation and therefore suitable for ARDS treatment, but more studies of the molecular mechanisms that determine surfactant resistance to inactivation are required in order to design resistant surfactant preparations in a rational manner. The rSP-C33Leu surfactant preparation we use herein contains an SP-C analogue but lacks an SP-B analogue and also the phospholipid composition differs from the one of paractant alfa; these differences may affect the spreading and function as well as resistance to inactivation.

## Conclusion

The pathogenesis of the early exsudative phase of ARDS includes not only surfactant dysfunction, but also prominent aspects of inflammation, vascular dysfunction, oxidant injury, cellular injury, and oedema. Herein, we present a two-hit rabbit model of ARDS that recapitulates the prominent features of the disease. We show that administration of rSP-C33Leu surfactant in this model results in improved lung functions, decreased oedema formation, and reduced pulmonary inflammation to almost the same degree as poractant alfa. Hence, the rSP-C33Leu surfactant used herein could potentially be developed further to increase its resistance to inactivation. For this purpose, the scalable recombinant rSP-C33Leu production process [40], and the possibility to generate mixtures with different phospholipid compositions are advantageous.

## Supplementary information

**Additional file 1: Table S1.** One-way ANOVA with Tukey post-hoc test to test the differences between the groups in the parameters with dynamic changes for fixed timepoint. Timepoints: before (basal value, BV) and after induced ARDS and within 3 h after administration of the therapy. Variables: Static lung-thorax compliance (Cstat), dynamic lung-thorax compliance (C_dyn_), the ratio of arterial oxygen partial pressure to fraction of inspired oxygen (P/F), oxygenation index (OI), airway pressure (Paw), alveolar-arterial gradient (AaG), ventilation efficiency index (VEI), partial pressure of oxygen (PaO_2_), partial pressure of carbon dioxide (PaCO_2_), oxygen saturation (SaO_2_), and arterial pH. CI denotes a 95% confidence interval for the difference of the population means.

**Additional file 2: Table S2.** The estimates of the trend (for each treatment) and *p* values using the linear mixed model with the fixed effect of time, drug and their interaction and the random effect of subjects. Dependent variables: Static lung-thorax compliance (C_stat_), dynamic lung-thorax compliance (C_dyn_), the ratio of arterial oxygen partial pressure to fraction of inspired oxygen (P/F), oxygenation index (OI), airway pressure (Paw), alveolar-arterial gradient (AaG), ventilation efficiency index (VEI), partial pressure of oxygen (PaO_2_), partial pressure of carbon dioxide (PaCO_2_), oxygen saturation (SaO_2_), and arterial pH. Estimates denotes the estimate of regression line slope. For poractant alfa and rSP-C33Leu the estimates are expressed relative to respective estimate of slope in control group. **Table S3.** The estimates of the difference of means and corrected *p* values using multiple comparisons of the means of a variable between drug treatments using the linear mixed model. Variables: Static lung-thorax compliance (C_stat_), dynamic lung-thorax compliance (Cdyn), the ratio of arterial oxygen partial pressure to fraction of inspired oxygen (P/F), oxygenation index (OI), airway pressure (Paw), alveolar-arterial gradient (AaG), ventilation efficiency index (VEI), partial pressure of oxygen (PaO2), partial pressure of carbon dioxide (PaCO2), oxygen saturation (SaO2), and arterial pH.

## Data Availability

The datasets used and/or analysed during the current study are available from the corresponding author on reasonable request.

## Abbreviations

AaG: Alveolar-arterial gradient
ARDS: Acute respiratory distress syndrome
C_dyn_: Dynamic compliance
C_stat_: Static compliance
OI: Oxygenation index
Paw: Airway pressure
PEEP: Positive end-expiratory pressure
PIP: Peak inspiratory pressure
P_plateau_: Plateau pressure
RDS: Respiratory distress syndrome
rSP-C33Leu: Recombinant surfactant protein C analogue
SP: Surfactant protein
sPLA2: Secretory phospholipase A2
VEI: Ventilation efficiency index
VILI: Ventilator-induced lung injury
V_T_: Tidal volume
