# Supplementary material for: Synthetic surfactant with a recombinant surfactant protein C analogue improves lung function and attenuates inflammation in a model of acute respiratory distress syndrome in adult rabbits
Source: Respir Res. 2019 Nov 6;20:245. doi: 10.1186/s12931-019-1220-x (PMC6836435; doi:10.1186/s12931-019-1220-x)
Supplement: Supplementary file 1 — Additional file 1: Table S1. One-way ANOVA with Tukey post-hoc test to test the differences between the groups in the parameters with dynamic changes for fixed timepoint. Timepoints: before (basal value, BV) and after induced ARDS and within 3 h after administration of the therapy. Variables: Static lung-thorax compliance (Cstat), dynamic lung-thorax compliance (Cdyn), the ratio of arterial oxygen partial pressure to fraction of inspired oxygen (P/F), oxygenation index (OI), airway pressure (Paw), alveolar-arterial gradient (AaG), ventilation efficiency index (VEI), partial pressure of oxygen (PaO2), partial pressure of carbon dioxide (PaCO2), oxygen saturation (SaO2), and arterial pH. CI denotes a 95% confidence interval for the difference of the population means. [file 12931_2019_1220_MOESM1_ESM.docx]

**Table S1** One-way ANOVA with Tukey post-hoc test to test the differences between the groups in the parameters with dynamic changes for fixed timepoint. Timepoints: before (basal value, BV) and after induced ARDS and within 3 h after administration of the therapy. Variables: Static lung-thorax compliance (Cstat), dynamic lung-thorax compliance (C_dyn_), the ratio of arterial oxygen partial pressure to fraction of inspired oxygen (P/F), oxygenation index (OI), airway pressure (Paw), alveolar-arterial gradient (AaG), ventilation efficiency index (VEI), partial pressure of oxygen (PaO_2_), partial pressure of carbon dioxide (PaCO_2_), oxygen saturation (SaO_2_), and arterial pH. CI denotes a 95% confidence interval for the difference of the population means.

|  |  | **Control *vs.* Poractant alfa** | | **Control *vs.* rSP-C33Leu** | | **Poractant alfa *vs.* rSP-C33Leu** | | **Effect size** |
| --- | --- | --- | --- | --- | --- | --- | --- | --- |
|  |  | ***p* value** | **CI** | *p* value | **CI** | ***p* value** | **CI** | **η^2^ value** |
| **C_stat_** | BV | 0.936 | (-0.70, 0.53) | 0.985 | (-0.66, 0.58) | 0.982 | (-0.57, 0.66) | 0.007 |
|  | ARDS | 0.237 | (-0.10, 0.50) | 0.165 | (-0.08, 0.53) | 0.973 | (-0.27, 0.33) | 0.195 |
|  | 0.5h | **0.048** | (0.00, 0.96) | 0.175 | (-0.13, 0.83) | 0.767 | (-0.61, 0.35) | 0.282 |
|  | 1h | 0.063 | (-0.02, 0.81) | 0.951 | (-0.47, 0.37) | **0.035** | (-0.86, -0.03) | 0.333 |
|  | 1.5h | **0.016** | (0.09, 0.89) | 0.462 | (-0.21, 0.59) | 0.165 | (-0.70, 0.10) | 0.355 |
|  | 2h | **0.001** | (0.18, 0.69) | **0.007** | (0.10, 0.61) | 0.698 | (-0.34, 0.18) | 0.540 |
|  | 2.5h | **0.031** | (0.04, 0.86) | 0.137 | (-0.09, 0.74) | 0.726 | (-0.54, 0.29) | 0.315 |
|  | 3h | **0.016** | (0.07, 0.74) | 0.117 | (-0.06, 0.61) | 0.580 | (-0.47, 0.20) | 0.359 |
| **C_dyn_** | BV | 0.816 | (-0.43, 0.71) | 0.983 | (-0.53, 0.61) | 0.895 | (-0.65, 0.45) | 0.020 |
|  | ARDS | 0.220 | (-0.07, 0.36) | 0.236 | (-0.07, 0.35) | 0.999 | (-0.21, 0.20) | 0.160 |
|  | 0.5h | **0.004** | (0.13, 0.68) | 0.433 | (-0.14, 0.42) | 0.050 | (-0.54, 0.00) | 0.419 |
|  | 1h | **0.010** | (0.08, 0.61) | 0.990 | (-0.25, 0.28) | **0.011** | (-0.58, -0.07) | 0.420 |
|  | 1.5h | **0.005** | (0.12, 0.66) | 0.460 | (-0.14, 0.40) | 0.055 | (-0.52, 0.00) | 0.408 |
|  | 2h | **0.002** | (0.13, 0.60) | **0.037** | (0.01, 0.48) | 0.389 | (-0.35, 0.11) | 0.448 |
|  | 2.5h | **0.005** | (0.11, 0.67) | 0.092 | (-0.03, 0.52) | 0.368 | (-0.42, 0.12) | 0.390 |
|  | 3h | **0.019** | (0.05, 0.60) | 0.373 | (-0.13, 0.43) | 0.237 | (-0.44, 0.09) | 0.312 |
| **P/F** | BV | 0.395 | (-19.56, 6.08) | 0.997 | (-13.21, 12.42) | 0.414 | (-6.04, 18.72) | 0.104 |
|  | ARDS | 0.909 | (-5.43, 7.58) | 0.595 | (-3.97, 9.04) | 0.829 | (-4.83, 7.75) | 0.047 |
|  | 0.5h | **0.002** | (10.54, 46.19) | **0.004** | (8.30, 43.96) | 0.942 | (-19.46, 14.99) | 0.495 |
|  | 1h | **0.023** | (3.80, 55.59) | 0.144 | (-5.68, 46.11) | 0.611 | (-34.49, 15.53) | 0.303 |
|  | 1.5h | **0.000** | (19.75, 55.59) | **0.030** | (1.70, 37.54) | **0.040** | (-35.36, -0.74) | 0.586 |
|  | 2h | **0.000** | (18.50, 50.27) | **0.002** | (8.57, 40.35) | 0.254 | (-25.27, 5.42) | 0.610 |
|  | 2.5h | **0.000** | (21.08, 52.46) | **0.005** | (6.83, 38.21) | 0.068 | (-29.41, 0.91) | 0.639 |
|  | 3h | **0.000** | (18.15, 49.67) | **0.014** | (3.66, 35.19) | 0.064 | (-29.72, 0.74) | 0.597 |
| **OI** | BV | 0.579 | (-0.26, 0.60) | 0.992 | (-0.45, 0.41) | 0.484 | (-0.60, 0.22) | 0.075 |
|  | ARDS | 0.979 | (-5.45, 4.66) | 0.785 | (-6.39, 3.72) | 0.879 | (-5.82, 3.94) | 0.023 |
|  | 0.5h | **0.000** | (-15.62, -4.81) | **0.000** | (-15.30, -4.49) | 0.987 | (-4.90, 5.54) | 0.591 |
|  | 1h | **0.006** | (-13.28, -2.13) | **0.019** | (-12.16, -1.00) | 0.859 | (-4.27, 6.51) | 0.411 |
|  | 1.5h | **0.000** | (-17.97, -7.10) | **0.000** | (-15.91, -5.03) | 0.588 | (-3.19, 7.32) | 0.657 |
|  | 2h | **0.000** | (-19.60, -7.22) | **0.000** | (-18.56, -6.17) | 0.899 | (-4.94, 7.03) | 0.645 |
|  | 2.5h | **0.000** | (-17.98, -7.46) | **0.000** | (-16.66, -6.14) | 0.791 | (-3.76, 6.40) | 0.689 |
|  | 3h | **0.000** | (-16.63, -6.75) | **0.000** | (-15.31, -5.43) | 0.766 | (-3.45, 6.09) | 0.678 |
| **Paw** | BV | 0.427 | (-0.09, 0.03) | 0.753 | (-0.08, 0.05) | 0.839 | (-0.05, 0.07) | 0.075 |
|  | ARDS | 0.994 | (-0.10, 0.11) | 0.909 | (-0.09, 0.12) | 0.944 | (-0.09, 0.11) | 0.010 |
|  | 0.5h | **0.012** | (-0.60, -0.07) | 0.073 | (-0.51, 0.02) | 0.641 | (-0.16, 0.35) | 0.353 |
|  | 1h | **0.001** | (-0.67, -0.19) | **0.039** | (-0.49, -0.01) | 0.151 | (-0.05, 0.41) | 0.508 |
|  | 1.5h | **0.002** | (-0.60, -0.14) | **0.017** | (-0.51, -0.05) | 0.582 | (-0.13, 0.31) | 0.463 |
|  | 2h | **0.001** | (-0.58, -0.14) | **0.004** | (-0.53, -0.10) | 0.839 | (-0.16, 0.26) | 0.508 |
|  | 2.5h | **0.002** | (-0.65, -0.15) | **0.002** | (-0.65, -0.15) | 0.999 | (-0.24, 0.24) | 0.510 |
|  | 3h | **0.002** | (-0.61, -0.13) | **0.004** | (-0.59, -0.11) | 0.973 | (-0.21, 0.25) | 0.490 |
| **AaG** | BV | 0.367 | (-3.30, 11.24) | 0.957 | (-8.34, 6.67) | 0.238 | (-12.07, 2.46) | 0.148 |
|  | ARDS | 0.967 | (-7.27, 5.98) | 0.807 | (-8.53, 5.18) | 0.15 | (-7.67, 5.58) | 0.021 |
|  | 0.5h | **0.002** | (-45.82, -10.19) | **0.004** | (-45.23, -8.43) | 0.985 | (-16.64, 18.99) | 0.508 |
|  | 1h | **0.020** | (-53.35, -4.22) | 0.107 | (-46.81, 3.93) | 0.732 | (-17.22, 31.91) | 0.330 |
|  | 1.5h | **0.000** | (-54.28, -20.37) | **0.021** | (-37.97, -2.95) | 0.051 | (-0.09, 33.82) | 0.622 |
|  | 2h | **0.000** | (-47.83, -18.60) | **0.001** | (-39.84, -9.65) | 0.325 | (-6.14, 23.09) | 0.650 |
|  | 2.5h | **0.000** | (-48.92, -21.11) | **0.002** | (-37.33, -8.61) | 0.097 | (-1.86, 25.95) | 0.687 |
|  | 3h | **0.000** | (-46.26, -17.66) | **0.008** | (-34.49, -4.95) | 0.102 | (-2.06, 26.54) | 0.631 |
| **VEI** | BV | 0.998 | (-5.99, 5.68) | 0.802 | (-4.52, 7.54) | 0.753 | (-4.17, 7.50) | 0.032 |
|  | ARDS | 0.783 | (-4.80, 8.27) | 0.791 | (-4.84, 8.23) | 0.999 | (-6.35, 6.28) | 0.028 |
|  | 0.5h | 0.150 | (-1.82, 14.14) | 0.774 | (-5.81, 10.15) | 0.406 | (-11.70, 3.72) | 0.166 |
|  | 1h | 0.139 | (-1.20, 10.21) | 0.893 | (-4.68, 6.73) | 0.270 | (-8.99, 2.03) | 0.184 |
|  | 1.5h | 0.144 | (1.34, 10.93) | 0.757 | (-4.40, 7.87) | 0.408 | (-8.99, 2.87) | 0.169 |
|  | 2h | **0.044** | (0.15, 11.25) | 0.233 | (-1.84, 9.26) | 0.624 | (-7.35, 3.38) | 0.256 |
|  | 2.5h | 0.058 | (-0.15, 10.49) | 0.107 | (-0.82, 9.82) | 0.942 | (-5.81, 4.47) | 0.259 |
|  | 3h | 0.059 | (-0.15, 9.41) | 0.267 | (-1.75, 7.81) | 0.661 | (-6.21, 3.02) | 0.235 |
| **PaO_2_** | BV | 0.394 | (-13.70, 4.25) | 0.996 | (-9.26, 8.69) | 0.414 | (-13.11, 4.23) | 0.104 |
|  | ARDS | 0.908 | (-5.43, 7.59) | 0.592 | (-3.97, 9.05) | 0.828 | (-7.75, 4.83) | 0.047 |
|  | 0.5h | **0.002** | (10.55, 46.21) | **0.004** | (8.31, 43.97) | 0.942 | (-14.99,19.46) | 0.495 |
|  | 1h | **0.023** | (3.81, 55.60) | 0.144 | (-5.67, 46.12) | 0.611 | (-15.54, 34.50) | 0.303 |
|  | 1.5h | **0.000** | (19.75, 55.60) | **0.030** | (1.70, 37.55) | **0.040** | (0.74, 35.36) | 0.586 |
|  | 2h | **0.000** | (18.50, 50.27) | **0.002** | (8.57, 40.35) | 0.254 | (-5.42, 25.27) | 0.610 |
|  | 2.5h | **0.000** | (21.09, 52.47) | **0.005** | (6.84, 38.22) | 0.068 | (-0.91, 29.41) | 0.640 |
|  | 3h | **0.000** | (18.15, 49.69) | **0.014** | (3.67, 35.20) | 0.064 | (-0.74, 29.72) | 0.598 |
| **PaCO_2_** | BV | 0.739 | (-1.45, 2.66) | 0.677 | (-2.75, 1.36) | 0.247 | (-3.28, 0.69) | 0.12 |
|  | ARDS | 0.801 | (-1.74, 1.04) | 0.550 | (-1.97, 0.81) | 0.901 | (-1.57, 1.11) | 0.053 |
|  | 0.5h | 0.830 | (-1.59, 1.00) | 0.373 | (-2.00, 0.59) | 0.697 | (-1.65, 0.85) | 0.087 |
|  | 1h | 0.490 | (-2.34, 0.87) | 0.114 | (-2.94, 0.27) | 0.597 | (-2.15, 0.95) | 0.182 |
|  | 1.5h | 0.903 | (-1.92, 1.36) | 0.824 | (-2.02, 1.25) | 0.984 | (-1.69, 1.48) | 0.018 |
|  | 2h | 0.265 | (-2.40, 0.53) | 0.070 | (-2.84, 0.10) | 0.719 | (-1.86, 0.98) | 0.224 |
|  | 2.5h | 0.175 | (-3.33, 0.51) | 0.104 | (-3.55, 0.28) | 0.951 | (-2.07, 1.63) | 0.211 |
|  | 3h | 0.086 | (-3.32, 0.19) | 0.087 | (-3.32, 0.20) | 0.999 | (-1.69, 1.70) | 0.249 |
| **SaO_2_** | BV | 0.155 | (-4.82, 0.64) | 0.269 | (-4.45, 1.00) | 0.936 | (-2.27, 3.00) | 0.173 |
|  | ARDS | 0.569 | (-11.63, 4.91) | 0.999 | (-8,23, 8.31) | 0.539 | (-4.59, 11.39) | 0.070 |
|  | 0.5h | 0.675 | (-3.24, 6.54) | 0.760 | (-3.53, 6.26) | 0.987 | (-5.01, 4.44) | 0.039 |
|  | 1h | 0.504 | (-4.12, 10.84) | 0.487 | (-4.04, 10.93) | 0.999 | (-7.14, 7.32) | 0.079 |
|  | 1.5h | 0.103 | (-1.07, 13.58) | 0.058 | (-0.21, 14.44) | 0.949 | (-6.21, 7.94) | 0.261 |
|  | 2h | **0.027** | (1.06, 18.88) | **0.017** | (1.78, 19.60) | 0.975 | (-7.88, 9.33) | 0.360 |
|  | 2.5h | 0.067 | (-0.58, 19.46) | **0.048** | (0.07, 20.11) | 0.984 | (-9.03, 10.33) | 0.285 |
|  | 3h | **0.008** | (4.03, 27.38) | **0.005** | (4.95, 28.30) | 0.977 | (-10.35, 12.20) | 0.445 |
| **pH** | BV | 0.973 | (-0.14, 0.11) | 0.891 | (-0.10, 0.15) | 0.763 | (-0.09, 0.15) | 0.025 |
|  | ARDS | 0.537 | (-0.07, 0.17) | 0.833 | (-0.15, 0.09) | 0.224 | (-0.19, 0.04) | 0.132 |
|  | 0.5h | 0.328 | (-0.03, 0.13) | 0.999 | (-0.08, 0.08) | 0.323 | (-0.13, 0.03) | 0.127 |
|  | 1h | 0.127 | (-0.02, 0.16) | 0.739 | (-0.06, 0.12) | 0.389 | (-0.13, 0.04) | 0.178 |
|  | 1.5h | 0.191 | (-0.02, 0.15) | 0.999 | (-0.08, 0.09) | 0.182 | (-0.14, 0.02) | 0.184 |
|  | 2h | 0.065 | (-0.00, 0.16) | 0.487 | (-0.04, 0.12) | 0.425 | (-0.12, 0.04) | 0.224 |
|  | 2.5h | 0.050 | (0.00, 0.25) | 0.291 | (-0.05, 0.20) | 0.572 | (-0.17, 0.07) | 0.245 |
|  | 3h | 0.057 | (0.00, 0.30) | 0.291 | (-0.06, 0.25) | 0.616 | (-0.20, 0.09) | 0.235 |
