# Supplementary material for: Synthetic surfactant with a recombinant surfactant protein C analogue improves lung function and attenuates inflammation in a model of acute respiratory distress syndrome in adult rabbits
Source: Respir Res. 2019 Nov 6;20:245. doi: 10.1186/s12931-019-1220-x (PMC6836435; doi:10.1186/s12931-019-1220-x)
Supplement: Supplementary file 2 — Additional file 2: Table S2. The estimates of the trend (for each treatment) and p values using the linear mixed model with the fixed effect of time, drug and their interaction and the random effect of subjects. Dependent variables: Static lung-thorax compliance (Cstat), dynamic lung-thorax compliance (Cdyn), the ratio of arterial oxygen partial pressure to fraction of inspired oxygen (P/F), oxygenation index (OI), airway pressure (Paw), alveolar-arterial gradient (AaG), ventilation efficiency index (VEI), partial pressure of oxygen (PaO2), partial pressure of carbon dioxide (PaCO2), oxygen saturation (SaO2), and arterial pH. Estimates denotes the estimate of regression line slope. For poractant alfa and rSP-C33Leu the estimates are expressed relative to respective estimate of slope in control group. Table S3. The estimates of the difference of means and corrected p values using multiple comparisons of the means of a variable between drug treatments using the linear mixed model. Variables: Static lung-thorax compliance (Cstat), dynamic lung-thorax compliance (Cdyn), the ratio of arterial oxygen partial pressure to fraction of inspired oxygen (P/F), oxygenation index (OI), airway pressure (Paw), alveolar-arterial gradient (AaG), ventilation efficiency index (VEI), partial pressure of oxygen (PaO2), partial pressure of carbon dioxide (PaCO2), oxygen saturation (SaO2), and arterial pH. [file 12931_2019_1220_MOESM2_ESM.docx]

**Table S2** The estimates of the trend (for each treatment) and *p* values using the linear mixed model with the fixed effect of time, drug and their interaction and the random effect of subjects. Dependent variables: Static lung-thorax compliance (C_stat_), dynamic lung-thorax compliance (C_dyn_), the ratio of arterial oxygen partial pressure to fraction of inspired oxygen (P/F), oxygenation index (OI), airway pressure (Paw), alveolar-arterial gradient (AaG), ventilation efficiency index (VEI), partial pressure of oxygen (PaO_2_), partial pressure of carbon dioxide (PaCO_2_), oxygen saturation (SaO_2_), and arterial pH. Estimates denotes the estimate of regression line slope. For poractant alfa and rSP-C33Leu the estimates are expressed relative to respective estimate of slope in control group.

|  |  |  |  |  |  |  |
| --- | --- | --- | --- | --- | --- | --- |
|  | Control | | Poractant alfa (relative to control) | | rSP-C33Leu (relative to control) | |
|  | Estimate | *p* value | Estimate | *p* value | Estimate | *p* value |
| C_stat_ | -0.18 | 0.000 | -0.02 | 0.817 | 0.05 | 0.427 |
| C_dyn_ | -0.15 | 0.000 | -0.01 | 0.740 | 0.05 | 0.272 |
| P/F | -0.22 | 0.910 | 2.61 | 0.337 | -1.24 | 0.646 |
| OI | 0.96 | 0.073 | -1.33 | 0.068 | -1.07 | 0.141 |
| Paw | 0.05 | 0.082 | -0.00 | 0.923 | -0.06 | 0.132 |
| AaG | -0.51 | 0.787 | -1.96 | 0.449 | 1.52 | 0.569 |
| VEI | -2.16 | 0.000 | -0.27 | 0.642 | 0.96 | 0.104 |
| PaO_2_ | -0.23 | 0.909 | -1.24 | 0.647 | 2.61 | 0.337 |
| PaCO_2_ | 0.59 | 0.001 | -0.52 | **0.035** | -0.35 | 0.147 |
| SaO_2_ | -5.10 | 0.000 | 5.27 | **0.002** | 5.70 | **0.001** |
| pH | -0.06 | 0.000 | 0.04 | 0.075 | 0.04 | 0.089 |

**Table S3** The estimates of the difference of means and corrected *p* values using multiple comparisons of the means of a variable between drug treatments using the linear mixed model.Variables: Static lung-thorax compliance (C_stat_), dynamic lung-thorax compliance (Cdyn), the ratio of arterial oxygen partial pressure to fraction of inspired oxygen (P/F), oxygenation index (OI), airway pressure (Paw), alveolar-arterial gradient (AaG), ventilation efficiency index (VEI), partial pressure of oxygen (PaO2), partial pressure of carbon dioxide (PaCO2), oxygen saturation (SaO2), and arterial pH.

|  |  |  |  |  |  |  |
| --- | --- | --- | --- | --- | --- | --- |
|  | Control *vs.* Poractant alfa | | Control *vs.* rSP-C33Leu | | Poractant alfa *vs.* rSP-C33Leu | |
|  | Estimate | *p* value | Estimate | *p* value | Estimate | *p* value |
| C_stat_ | 0.47 | **0.009** | 0.15 | 0.343 | -0.32 | 0.066 |
| C_dyn_ | 0.40 | **0.000** | 0.07 | 0.492 | -0.33 | **0.001** |
| P/F | 28.90 | **0.001** | 24.24 | **0.003** | -4.66 | 0.539 |
| OI | -9.05 | **0.000** | -8.31 | **0.000** | 0.74 | 0.719 |
| Paw | -0.37 | **0.000** | -0.21 | **0.026** | 0.16 | **0.049** |
| AaG | -28.95 | **0.000** | -28.36 | **0.002** | 3.59 | 0.632 |
| VEI | 5.64 | 0.054 | 1.022 | 0.668 | -4.61 | 0.068 |
| PaO_2_ | 24.25 | **0.003** | 28.91 | **0.001** | 4.66 | 0.539 |
| PaCO_2_ | 0.03 | 0.955 | -0.55 | 0.481 | -0.58 | 0.481 |
| SaO_2_ | -1.49 | 0.673 | -1.76 | 0.673 | -0.27 | 0.888 |
| pH | 0.02 | 0.556 | -0.03 | 0.556 | -0.05 | 0.556 |
